# Dosimetric impact of variable bladder filling on IMRT planning for locally advanced carcinoma cervix

**DOI:** 10.1186/s43046-020-00033-5

**Published:** 2020-07-31

**Authors:** Soumya Dutta, Abhinav Dewan, Swarupa Mitra, Manoj Kumar Sharma, Sumeet Aggarwal, Soumitra Barik, M. Mahammood Suhail, Maninder Bhushan, Anurag Sharma, Inderjeet Kaur Wahi, Kiran Dobriyal, Jwala Mukhee

**Affiliations:** 1 Department of Radiotherapy, Rajiv Gandhi Cancer Institute and Research Centre, Sector-5, Rohini, New Delhi, India; 2 Department of Radiotherapy, Fortis Hospital, Noida, Uttar Pradesh India; 3 Department of Medical Physics, Rajiv Gandhi Cancer Institute and Research Centre, New Delhi, India; 4 Department of Medical Statistics, Rajiv Gandhi Cancer Institute and Research Centre, New Delhi, India

**Keywords:** Variable bladder filling, Locally advanced cervical cancer, IMRT

## Abstract

**Background:**

To evaluate the dosimetric impact of variable bladder filling on target and organ at risk (OARs) in cervical cancer patients undergoing chemoradiation. Forty consecutive patients with cervical cancer underwent radiotherapy planning as per the departmental protocol. All patients were asked to empty their bowel and bladder before simulation and catheterization was done. Normal saline was instilled into the bladder through Foleys till the patient had a maximal urge to urinate. Pelvic cast fabrication and CT simulation was done.

Then, 30%, 50%, and 100% of the instilled saline was removed and rescans taken. Planning was done on full bladder (X) and the same plan applied to the contours with bladder volumes 0.7X (PLAN70), 0.5X (PLAN50), and empty (PLAN0). A dose of 50 Gy/25# was prescribed to the PTV and plans evaluated. Intensity-modulated radiotherapy plans with full bladder were implemented for each patient. Shifts in the center of mass (COM) of the cervix/uterus with variable bladder filling identified were noted. Statistical analysis was performed using SPSS software. A *p* value < 0.05 was considered significant.

**Results:**

Bladder volume in 70%, 50%, and empty bladder planning was 78.34% (388.35 + 117.44 ml), 64.44% (320.60 + 106.20 ml), and 13.63% (62.60 + 23.12 ml), respectively. The mean dose received by 95% PTV was 49.76 Gy + 1.30 Gy. Though the difference in target coverage was significant between PLAN100 and other plans, the mean difference was minimal. A decrease in bladder filling resulted in an increase in OAR dose. Variation in the increase in dose to OARs was not significant if bladder filling was > 78.34% and > 64.44% of a full bladder with respect to the bowel and rectal/bladder doses, respectively. Inconsistent bladder filling led to a maximal shift in COM (uterus/cervix) in the *Y*- and *Z*-axis.

**Conclusion:**

Bladder filling variations have an impact on cervico-uterine motion/shape, thereby impacting the dose to the target and OARs. It is recommended to have a threshold bladder volume of at least 70–75% of optimally filled bladder during daily treatment.

**Trial registration:**

Institutional review board (IRB) registered by Drug Controller General (India) with registration number ECR/10/Ins/DC/2013. Trial Registration number - RGCIRC/IRB/44/2016, registered and approved on the 14th of May 2016.

## Background

Cervical cancer is the fourth most common cancer worldwide and is the fourth commonest cause of cancer-associated death among females [1]. Most of our cervical cancer patients present with a locally advanced disease. Concurrent chemoradiation (CTRT) plays an important role in the definitive treatment of these patients with intensity-modulated radiotherapy (IMRT) being used to intensify the target dose, while sparing adjacent normal tissues, thereby decreasing toxicity.

IMRT conforms high-dose region to the target volume (TV) and tends to have a strict CTV/PTV margin. However, this high conformal target does not take into account tumor regression and organ motion. These factors can lead to geographical miss and should be taken into consideration during daily treatment. Minor variation in target motion can lead to underdosing or delivery of excessive dose to organ at risk (OAR) as a result of its steep dose gradient [2].

Pelvic radiotherapy with a full bladder is presently followed worldwide with a rationale for better OAR sparing. However, unpredictability may occur due to variable bladder filling during treatment. This occurs due to patients’ poor compliance, anatomical alterations during radiotherapy or toxicity [3]. Variable bladder filling during pelvic radiotherapy similarly leads to displacement and modification in the size/shape of target and OARs, which may affect the treatment outcome. Understanding these factors is important and should be taken into consideration especially during IMRT treatment. It is necessary to deliver IMRT with the precise known bladder volume if possible [2]. Currently, bladder filling protocol is followed during radiotherapy planning with the aim of treating with comfortably full bladder [4]. Lack of adherence to bladder protocol by the patient in subsequent treatment days can cause the differential day to day bladder volumes, which may adversely impact the dose to TV and OARs. Therefore, the aim of the present study is to investigate the dosimetric impact of variable bladder filling on a deformable target volume and OARs in cervical cancer patients undergoing pelvic radiotherapy.

## Methods

Between June 2016 and June 2017, forty consecutive patients with locally advanced cervical carcinoma undergoing concurrent chemoradiation followed by brachytherapy were enrolled in a prospective observational study conducted in the Department of Radiotherapy at a tertiary cancer center in India. Eligibility criteria included (1) histologically proven squamous cell carcinoma of the cervix, (2) planned for definitive treatment with IMRT and concomitant chemotherapy followed by brachytherapy, (3) informed consent taken, (4) age 18–70 years, (5) Eastern Co-operative Oncology Group (ECOG) performance score of 0–2, and (6) no previous history of radiation. The study was approved by the ethics committee of the hospital.

Pretreatment evaluation was done by complete diagrammatic and descriptive documentation of the extent of the primary and regional disease, histopathology, blood tests, imaging, and cystoscopy/sigmoidoscopy when indicated and PET scan (in selected cases).

### Treatment planning

All patients underwent immobilization, CT simulation, and treatment planning as per the departmental protocol. Planning images were transferred to Varian Eclipse contouring station version 11. Target volume and OARs were contoured as per RTOG contouring guidelines [4]. GTV represented gross visible tumor and/or enlarged lymphnodes identified clinically or on imaging. CTV included regions at high risk of microscopic disease and was contoured as per the institutional guidelines. A differential PTV margin was generated to account for daily setup error and organ motion [5, 6]. OARs included the bowel, rectum, bladder, and femoral heads. For organs at risk, dose constraints prescribed were Cord Dmax < 45 Gy, bladder V45 Gy ≤ 35%, bowel V40 Gy ≤ 30%, rectum V30 Gy ≤ 60%, and bilateral femur V30 Gy ≤ 15% with minor deviation acceptable as per institutional protocol [7]. IMRT plans were generated and approved for each patient. All plans were normalized in order for 95% PTV to receive the prescribed dose.

All patients were asked to empty their bowels on the morning of the simulation. Before taking the patient on the couch, patients were asked to pass urine in order to evacuate the urinary bladder completely. Patient positioning was done on the CT simulator (Siemens Somatom Sensation) and catheterized with Foleys’ catheter. Residual urine was drained using the Foleys. Normal saline was instilled into the bladder through Foleys’ catheter till the patient had a maximal urge to urinate, and the amount of normal saline thus instilled was calculated (X ml). This was taken as 100% bladder fullness for that patient. Pelvic cast fabrication was done after obtaining appropriate immobilization in a supine position using a headrest and with the hands above the head. CT scan with intravenous contrast from T12 vertebral body to 5 cm below the ischial tuberosity with 3-mm-slice thickness was taken. The scan was named as “IMRT100% X ML”.

Then, 30% of the instilled normal saline was taken out and a scan was taken again at 70% full bladder. The scan was named as “IMRT70% 0.7X ML”.

Twenty percent of the instilled normal saline was further taken out and a repeat scan was taken. The scan was named as “IMRT50% 0.5X ML”.

Subsequently, all the normal saline was taken out and 0% bladder scan taken. The scan was named as “IMRT0% EMPTY”.

In this step, any extra fluid taken out other than the amount of normal saline instilled was noted (Y) and this would thus tell us about the amount of urine formed during CT simulation. Bladder volume at 70%, 50%, and an empty bladder radiotherapy plan was calculated by adding Y and 0.7X/0.5X/0X ml bladder volume.

Planning was then done on full bladder volume (X), and then, the same plan was applied to the contours with bladder volumes 0.7X, 0.5X, and empty bladder and named PLAN100, PLAN70, PLAN50, and PLAN0, respectively.

A dose of 50 Gy/25# (2 Gy/#) was delivered 5 days/week for five weeks along with concurrent weekly chemotherapy. Treatment plans were evaluated using the following dosimetric parameters:

1. D95% (dose received by 95% PTV volume)
2. *V*_50 Gy_ (% volume receiving 50 Gy dose) and *D*_mean_ of the urinary bladder and rectum
3. *V*_45 Gy_ and *D*_mean_ of the bowel
4. Homogeneity index (H.I.) and conformity index (C.I.)

The best plan for treatment was selected based on current guidelines of optimal urinary bladder filling that advocates’ use of comfortably filled bladder (100% full bladder) during pelvic radiotherapy. CBCT images were acquired and compared to the reference images to measure the bladder volume.

The center of mass (COM) of the cervix and uterus was identified on full bladder scan (PLAN100) (reference scan), and shifts in COM with variable bladder filling in terms of *x*- (lateral), *y*- (longitudinal), and *z*-axis (vertical) were noted. Association between the variable bladder filling and cervico-uterine axis shift was studied with an aim to estimate the cervico-uterine position and shape before daily radiotherapy fraction from the measured bladder volume.

All statistical analysis was performed using a statistical package for social sciences (Version 20, SPSS Inc, Chicago, IL, USA). Descriptive and dosimetric data was presented as a mean with a standard deviation (SD) and percentages for quantitative variables. Paired *t* test or Wilcoxon signed-rank test was used for comparison of mean between each plan depending upon the nature of data. Correlation between the change in bladder volume and utero-cervical motion along with dose in OARs was noted using Spearman/Pearson correlation test. A *p* value < 0.05 was considered statistically significant.

## Results

A total of forty patients with locally advanced carcinoma cervix were enrolled in the present study conducted at a tertiary cancer center in India. Patient and disease characteristics are summarized in Table 1. The mean age at diagnosis was 53.15 ± 10.61 (range, 30–79 years). The majority of the patients had stage IIB disease (42.50%).

**Table 1:** Patient and treatment characteristics of patients enrolled in the present study

Characteristics	***N*** (%)
**Age (mean+SD)**	53.15 ± 10.61 (range, 30–79 years)
**Parity (1:2:3:4:5:6)**	2(5%): 4(10%): 10(25%): 15(37.50%): 6(15%): 3(7.50%)
**ECOG performance status (0:1:2)**	5 (12.5%): 25 (63.5%): 10 (25%)
**FIGO tumor stage (IIA:IIB:IIIA:IIIB:IVA)**	4(10%): 17(42.50%): 4(10%): 14(35%): 1(2.50%)
**Grade (I:II:III)**	10(25%): 25(63.5%): 5(12.5%)
**Pelvic lymphnodes (present: absent)**	28 (70%): 12 (30%)
**Bulky disease (> 4 cm) (present: absent)**	30 (75%): 10 (25%)
**External beam radiotherapy dose (PTV D95%)**	Mean 49.76 Gy + 1.29 Gy (range, 44.91–51.33 Gy)
**Concurrent chemotherapy (cisplatin: carboplatin)**	32 (80%):8(20%)

Bladder volume at 70%, 50%, and in empty bladder radiotherapy planning was calculated taking into consideration the total amount of urine formed during the simulation and the amount of saline instilled for each radiotherapy plan. One hundred sixty CT scans were taken for radiotherapy planning for all the patients. Bladder volume for 70% (PLAN70), 50% (PLAN50), and empty bladder (PLAN0) corresponded to 78.34% (388.35 + 117.44 ml), 64.44% (320.60 + 106.20 ml), and 13.63% (62.60 + 23.12 ml) volume, respectively.

Dosimetric and volumetric parameters were compared and presented in Tables 2 and 3. The mean dose received by 95% PTV was 49.76 Gy ± 1.30 Gy (range, 48.55–51.33Gy). Though the difference in the target volume coverage was statistically significant between the full bladder (PLAN100) and other radiotherapy plans (*p* < 0.05, Table 3), the mean difference in coverage was minimal (< 1 Gy). No impact on target volume coverage was seen with a further decrease in urinary bladder filling.

**Table 2:** Dosimetric comparison between different radiotherapy plans

Treatment plans	PTV D95%(Gy)	Bladder ***V***_**50 Gy**_ (%)	Bladder mean (Gy)	Rectum ***V***_**50 Gy**_ (%)	Rectum mean (Gy)	Bowel ***V***_**45 Gy**_ (%)	Bowel mean (Gy)	H.I.	C.I.	Actual % bladder filling*
**PLAN100**	49.76 ± 1.30	38.65 ± 5.51	40.87 ± 2.05	28.50 ± 6.62	44.48 ± 2.04	0.26 ± 0.2	19.85 ± 2.76	0.077 ± 0.021	0.944 ± 0.088	100% (494.85 + 143.28 ml)
**PLAN70**	49.41 ± 1.21	39.86 ± 5.23	41.76 ± 1.98	28.41 ± 7.87	44.59 ± 2.49	0.84 ± 1.04	21.58 ± 3.06	0.091 ± 0.018	0.859 ± 0.07	78.34% (388.35 + 117.44 ml)
**PLAN50**	49.33 ± 1.24	40.78 ± 5.75	42.27 ± 2.11	28.24 ± 7.46	44.73 ± 2.89	0.89 ± 1.16	21.85 ± 2.99	0.097 ± 0.029	0.854 ± 0.77	64.44% (320.60 + 106.20 ml)
**PLAN0**	49.32 ± 1.18	41.45 ± 8.94	45.85 ± 2.26	30.05 ± 8.61	45.07 ± 2.82	1.04 ± 0.82	22.59 ± 2.85	0.094 ± 0.018	0.847 ± 0.07	13.63% (62.60 + 23.12 ml)

*Actual percentage bladder filling is different from the amount of fluid instilled by Foley’s catheter due to residual urine volume and amount of urine formed during the process of simulation

**Table 3:** *p* value of dosimetric variables on comparing different radiotherapy plans

Plan comparison	Rectum mean	Rectum ***V***_**45 Gy**_	Bladder mean	Bladder ***V***_**45 Gy**_	Bowel mean	Bowel ***V***_**45 Gy**_	PTV D95%
**PLAN100 vs. PLAN70**	0.697	0.941	**0.000**	0.190	**0.000**	**0.001**	**0.000**
**PLAN100 vs. PLAN50**	0.459	0.823	**0.000**	0.099	**0.000**	**0.001**	**0.000**
**PLAN100 vs. PLAN0**	0.088	0.289	**0.000**	0.134	**0.000**	**0.000**	**0.000**
**PLAN70 vs. PLAN50**	0.412	0.790	**0.001**	0.351	**0.010**	0.528	0.180
**PLAN70 vs. PLAN0**	0.060	0.111	**0.000**	0.343	**0.000**	0.102	0.110
**PLAN50 vs. PLAN0**	**0.037**	0.090	**0.000**	0.568	**0.001**	0.222	0.820

### Bladder dosimetry (Fig. 1)

There was no statistical difference in different bladder filling plans as far as urinary bladder *V*_50 Gy_ was concerned. However, the percentage increase in bladder *V*_50_ was less variable up to PLAN50 (64.44% bladder filling). A rapid increase in the *V*_50 Gy_ dose gradient was noted with a further decrease in bladder filling.

**Fig. 1:**
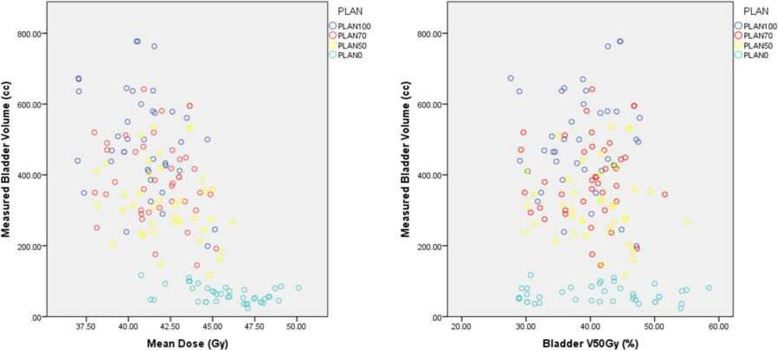
Scatterplot showing correlation between bladder volumes and corresponding mean dose and *V*_50 Gy_ of the bladder for different plans

Bladder mean dose showed a statistically significant increasing trend with an increase in bladder emptying (*p* < 0.05). Mean dose showed a gradually increasing slope till 64.44% bladder filling (PLAN50) after which it showed a rapid rise. A significant correlation between the percentage increase in the mean bladder dose with the decrease in bladder filling beyond 64.44% (PLAN50) was seen (*p* < 0.05).

### Rectal dosimetry (Fig. 2)

There was no statistical difference in between different bladder filling plans as far as rectal *V*_50 Gy_ was concerned. However, the percentage increase in the *V*_50 Gy_ was less up to PLAN50 (64.44% bladder filling) after which a rapid increase in the dose gradient was noted.

**Fig. 2:**
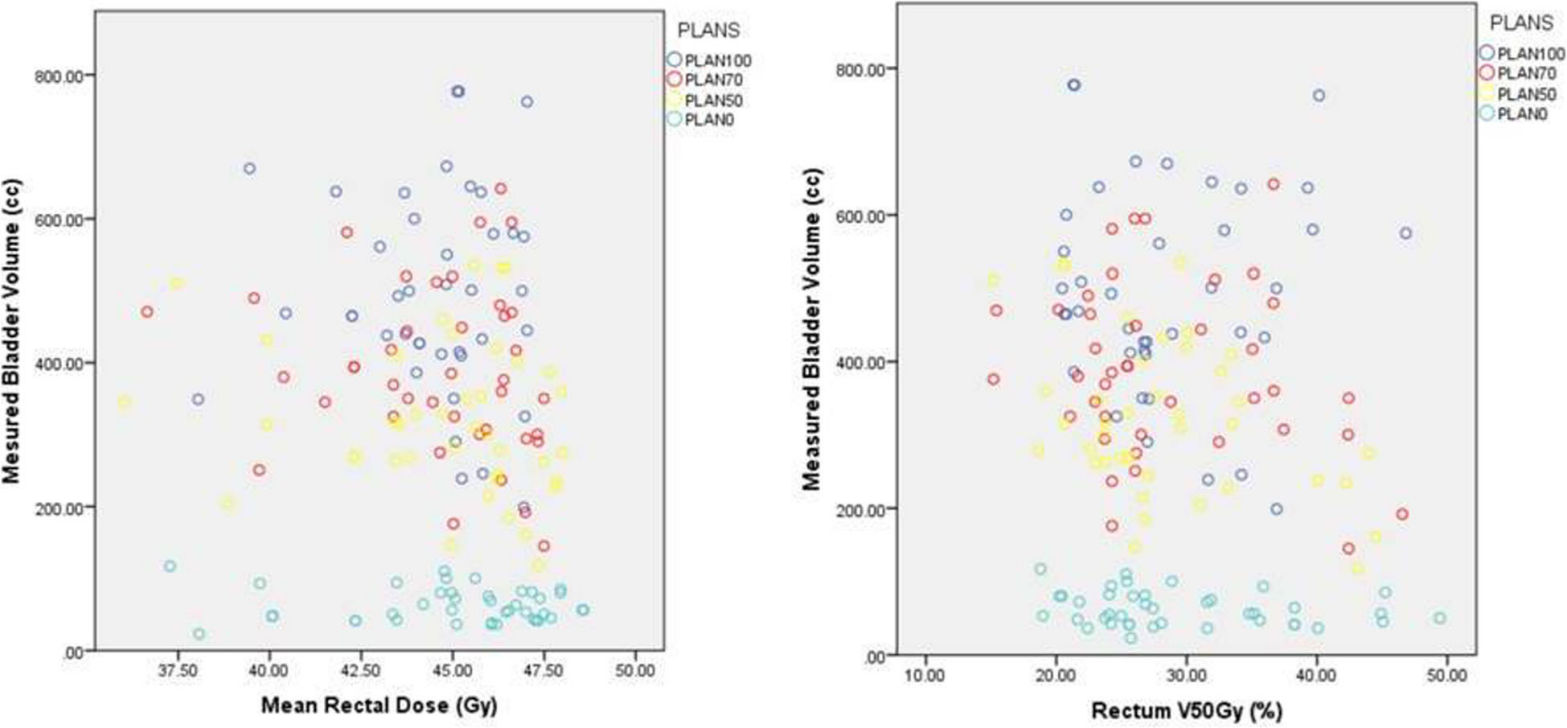
Scatterplot showing correlation between bladder volumes and corresponding mean dose and *V*_50 Gy_ of rectum for different plans

The mean rectal dose showed minimal variation with increased bladder emptying. Maximal variation of 1.33% (+ 4.78%) was noted between PLAN50 and empty bladder plan. Despite the minimal variation, a statistically significant difference in mean rectal dose was noted between PLAN50 and PLAN0 (*p* = 0.037).

### Bowel dose (Fig. 3)

Bowel *V*_45 Gy_ and mean dose showed a statistically significant increase in dose between PLAN100 vs. PLAN70, PLAN100 vs. PLAN50, and PLAN100 vs. PLAN0 (*p* < 0.05). However, a steep relative increase in bowel *V*_45 Gy_ and bowel mean was noted up to PLAN70 (78.34% bladder filling), after which the rate of increase slowed down. A significant correlation between the percentage increase in *V*_45 Gy_ bowel dose and percentage decrease in bladder filling in PLAN50 was seen (*p* = 0.008).

**Fig. 3:**
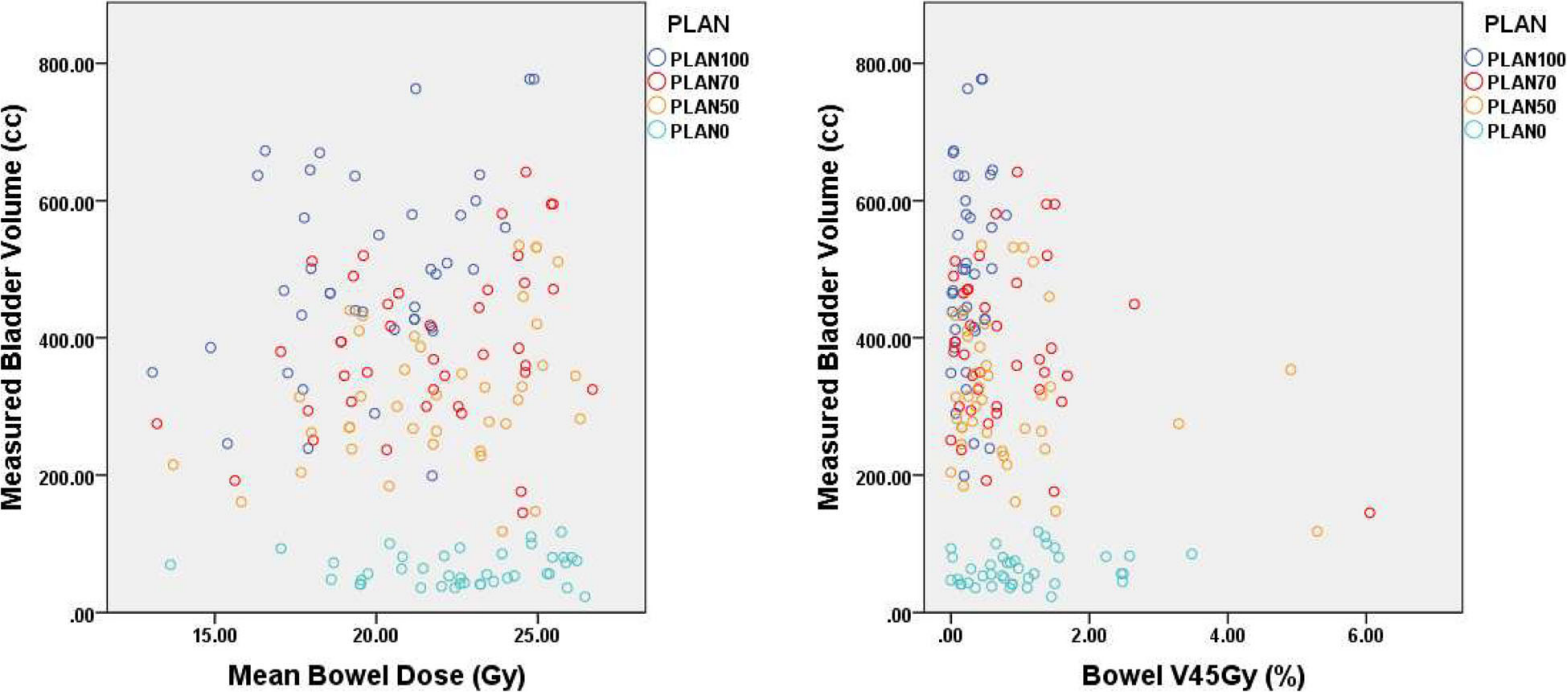
Scatterplot showing correlation between bladder volumes and corresponding mean dose and *V*_45 Gy_ of bowel for different plans

### Homogeneity and conformity

HI and CI showed a statistically significant deviation from optimal values on comparing PLAN70, PLAN50, and PLAN0 in comparison to full bladder radiotherapy plans. The percentage deviation from optimal value for HI and CI was maximal up to PLAN70 (78.34% bladder filling) and PLAN50 (64.44% bladder filling), respectively, after which uniform further deviation was seen.

Overall, a minimum of 64.44 to 78.34% bladder should be filled to obtain radiotherapy plans with adequate target volume coverage and minimal dose to OARs.

Variable bladder filling led to a shift in COM of utero-cervical axis (Table 4). The maximal shift in COM was noted in the *Y*-axis (superior-inferior axis) and *Z*-axis (antero-posterior axis) for all the plans. Correlation between the uterine shifts in *Y*-axis (correlation coefficient − 0.6, *p*< 0.005) and *Z*-axis (correlation coefficient − 0.50, *p*< 0.025) correlated significantly with an absolute change in the bladder volume between PLAN100 and PLAN50. The percentage decrease in bladder volume between PLAN100 and PLAN50 showed a significant correlation with the change in uterine *Z*-axis coordinates (correlation coefficient − 0.43, *p* = 0.05).

**Table 4:** Shifts in the center of mass (COM) of utero-cervical axis with variable bladder filling

Shifts	Differential bladder filling		
**Uterus**	**PLAN70**	**PLAN50**	**PLAN0**
*X*-axis^*^	− 0.11 + 0.45	− 0.13 + 0.54	− 0.14 + 0.69
*Y*-axis^^^	0.48 + 0.48	0.66 + 0.39	1.20 + 0.45
*Z*-axis^#^	− 0.15 + 0.20	− 0.37 + 0.27	− 0.64 + 0.42
**Cervix**	**PLAN70**	**PLAN50**	**PLAN0**
*X*-axis	− 0.22 + 0.25	− 0.33 + 0.31	0.05 + 0.51
*Y*-axis	0.41 + 0.64	0.47 + 0.45	0.52 + 0.58
*Z*-axis	− 0.10 + 0.21	− 0.26 + 0.29	− 0.41 + 0.27

*Left-right lateral

^Superior-inferior axis

^#^Antero-posterior axis

## Discussion

Bladder filling protocols are beneficial for radiotherapy planning of cervical cancers in terms of sparing OARs. Various studies have shown that during radiotherapy, bladder volumes obtained during initial planning scans differ from the bladder volumes that are being actually treated on a daily basis. This may be due to the fact that patients on radiotherapy are not able to maintain consistent bladder filling [8, 9]. Variability in bladder filling may lead to a lack of reliable dose constraints for pelvic OARs. The goal of the present study was to analyze the dosimetric impact of the variable bladder filling bladder (100%/70%/50%/EMPTY) on the target volume and OARs.

Variable bladder filling occurs as a result of a constant and dynamic process of urine formation. It depends upon the amount of water intake before bladder emptying, the rate of urine formation during simulation, and the amount of residual urine in the urinary bladder. The amount of saline instilled through the foleys after emptying the bladder as seen in our study is not an accurate depiction of the 100% actual urinary bladder filling “X” ml as there tends to be some residual urine present in the bladder. Likewise, the 70% and 50% bladder filling are not representative of actual bladder volume at that time of normal saline instillation since some urine is being formed during the cast and scan process. Similarly, the empty bladder has some residual urine left which also adds to the bladder volume. So to counter all these variables, the actual urinary bladder volumes of PLAN100, PLAN70, PLAN50, and PLAN0 were calculated from the planning CT scan images of each respective plan.

As in the present study, the maximum comfortable urinary bladder volume value of 100% was taken for each patient and the mean bladder volume was calculated in terms of percentage bladder filling to determine the dosimetrically crucial points beyond which a substantial change in dose to target or OARs was seen. The mean bladder volume for PLAN100, PLAN70, PLAN50, and PLAN0 differed significantly from each other (*p* < 0.05). Full bladder treatment can lead to 7 to 450% variation in the bladder volume signifying a low reliability despite instructions [10]. The literature review showed a decrease in bladder volume by 56% (*p* < 0.01) after 40 Gy of pelvic radiotherapy, thereby highlighting the need of adapting to bladder volume during treatment [4, 11]. Further studies confirm the above fact indicating a downward trend for the bladder volume during radiation in comparison to the initial value despite bladder protocols [12, 13].

IMRT technique aims to reduce normal tissue toxicity and provides adequate target volume coverage. Standard population-based margins are given to ensure adequate target coverage. However, these margins may not be sufficient due to the variability and extent of utero-cervical motion [11]. Variable bladder filling led to a considerable displacement of utero-cervical COM in the *Y*- and *Z*-axis without compromising the target volume coverage in the present study. In contrast, Jadon et al. [14] reviewed the literature regarding variable organ motions and concluded that a compromise on uterine coverage was noted with variable bladder volumes. Several studies have demonstrated the extent of target motion occurring during radiotherapy with the maximum reported displacement of uterine fundus up to 4.8 cm [15–19]. Huh et al. [15] and Lee et al. [16] compared the magnetic resonance images taken before and during pelvic radiotherapy and noted a significant change in the uterine movement and position due to variable bladder filling. Taylor et al. [17] made an assessment of the inter-fractional uterine and cervical motion in order to estimate the CTV to PTV margins. Taylor et al. noted a mean displacement of 0.70 cm and 0.71 cm in the antero-posterior (*Z*-axis) and superior-inferior (*Y*-axis) direction, respectively, which collaborated with our study findings. Often clinicians tend to take generous margins (2.5–4 cm) to account for 90% of cervico-uterine motion, thereby offsetting the benefit achieved with IMRT. These changes can be attributed not only to rectal and bladder filling variations, but can occur due to tumor regression [11, 17, 18, 20, 21]. In order to avoid excessive normal tissue irradiation, reduced margins can be used which can lead to underdosing in patients with large target movement and should thus be avoided.

Association of rectal and bladder filling with utero-cervical mobility has an attractive prospect for adaptive radiotherapy [2]. Despite using strict bladder and bowel protocol, large systematic setup errors especially at the fundus have been reported [2]. It has been observed that the bladder tends to change in shape and position during the course of pelvic radiotherapy [22, 23]. Huang et al. [22] and Roeske et al. [23] noted a 44% and 30% variation in bladder volume, respectively, during the course of fractionated pelvic radiotherapy and noted higher doses being delivered than initially calculated. Bladder volume reproducibility thus cannot be assured during pelvic radiotherapy treatment. Isodose levels as seen on the dose-volume histogram tend to indicate the planning CT scan state and are not relevant for the whole 5 weeks of treatment. Recent studies have also emphasized on the necessity for quicker bladder volume evaluation using either CBCT scans or ultrasonography in order to choose the most suitable plan of the day from the planning library for sparing OARs and ensuring precise radiation delivery [24].

A filled-up bladder is a principal reason for the dose sparing of the OARs concerned. The severity of acute and chronic effects on the bladder tends to increase after an integral radiotherapy dose of > 65 Gy (whole bladder) [25]. In our study, the bladder mean dose showed a slight, but statistically significant declining trend with increasing bladder volume which was in line with the previously published literature (*p* < 0.05) [18]. Completely filled up bladder decreased the integral bladder dose, while improved reproducibility was seen with the empty bladder at the cost of increased toxicity due to a larger bladder volume being in the high-dose region. Buchali et al. [18] therefore advocated the use of moderately filled bladder during treatment in order to avoid extreme departure from normal utero-cervical mobility. In contrast, the difference was not significant for *V*_50 Gy_ which can be ascertained to the fact that the filled-up bladder tends to move more superiorly and posteriorly, thereby extending into the PTV volume. This fact may be responsible for minimal difference in *V*_50 Gy_ value between different plans [19]. It may also indicate need for slightly less than full bladder filling which is a compromise between *D*_mean_ and *V*_50 Gy_ value. Also, a slow gradual rise up to 64.44% (PLAN50) bladder filling followed by a sudden rise in mean and *V*_50 Gy_ dose was noted. Therefore, at least 64.44% bladder filling was desirable at the time of daily treatment.

Bladder filling tends to displace the rectum outside the high-dose region. Rectal *V*_50 Gy_ and mean dose showed a minimal absolute change with an increase in bladder emptying. However, the relative increase in dose gradient was maximal between PLAN50 and PLAN0. So, a bladder filling volume at PLAN50 (64.44%) can be considered an optimal lower limit of bladder filling for rectal dosimetry.

**Fig. 4:**
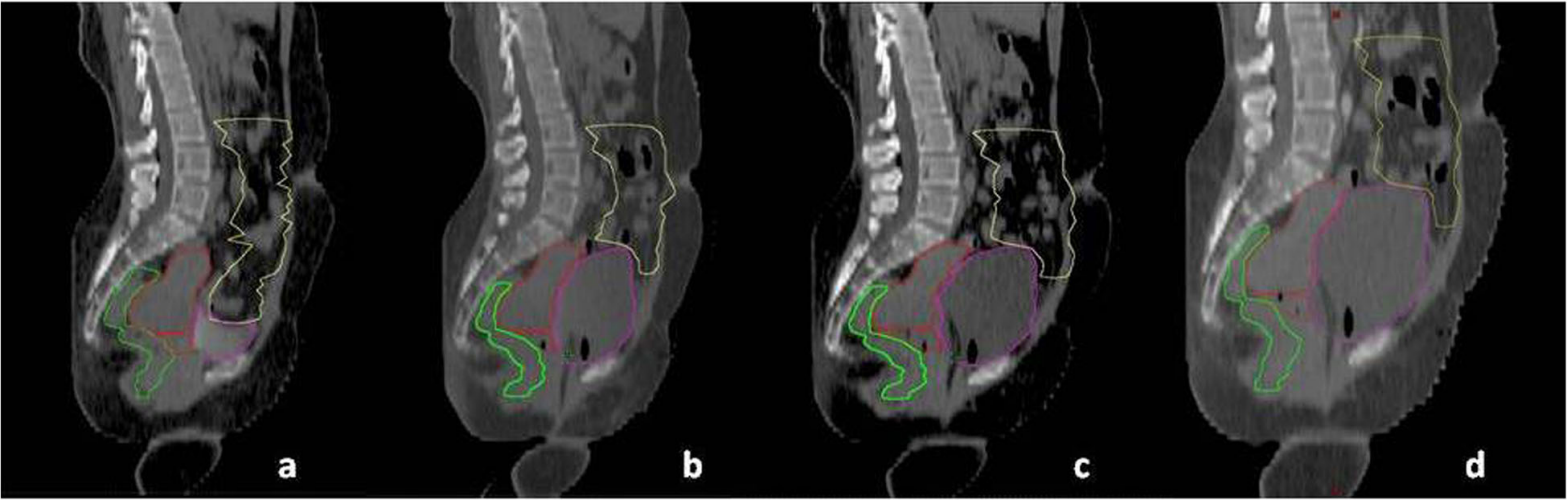
Showed an impact of bladder filling on small bowel (**a**) Empty bladder, (**b**) PLAN50, and (**c**) PLAN70 and (**d**) PLAN100

Published literature has documented bladder filling variation as an important contributing factor to bowel toxicity. It is therefore recommended to treat patients with a full bladder as it tends to push the small bowel outside the PTV target volume, thereby decreasing the bowel toxicity [4, 24–26]. As the bladder emptying increases, the bowel displaces into the pelvis and receives excess irradiation (Fig. 4). Yaparpalvi et al. [12] described the outcome as a result of variable bladder filling and noted a median superior-inferior bowel shift of 12.5 mm on an empty bladder when compared to a full bladder. This shift led to an extra 151 cc of bowel volume of being irradiated [12]. The mean dose and percentage of bowel receiving 45 Gy in our study showed a statistically significant difference between PLAN100 in comparison to other plans. Similar to our findings, treatment with full bladder reduced small bowel irradiation by 72% [27]. Simpson et al. [28] reported a 65% and 33% incidence of greater than or equal to grade 2 gastrointestinal toxicity in patients with bowel receiving *V*_45 Gy_ > 150 ml or below it, respectively. In addition, our results showed a sharp relative increase in dose gradient up to 78.34% bladder filling, after which the rate of increase slowed down. The percentage increase in *V*_45 Gy_ and a relative decrease in the bladder filling beyond PLAN50 were noted. In line with our study, a significant correlation between the volume of intestine irradiated and bladder volume was demonstrated by several studies [29]. Thus, a minimum of 64.44–78.34% bladder filling was desirable at the time of daily treatment in order to prevent excess bowel toxicity.

Homogeneity index (HI) is used to analyze as to how uniform the dose distribution and the target volume is, and a value close to zero is considered ideal [30]. HI showed an increasing trend with a decrease in bladder filling. The percentage difference in HI was most marked till 78.34% bladder filling (PLAN70). Additional parameters like conformity index (CI) were also used to analyze the treatment plans and value close to 1 is considered ideal [30]. The CI showed a decreasing trend with a decrease in bladder filling and maximum relative change noted till PLAN50. Thus, it can be considered that it is ideal to maintain a urinary bladder filling of at least 64.44% when compared to maximal comfortable urinary bladder filling.

Treatment with full bladder tends to induce large variations in inter-fractional bladder volume, leading to a potential for dosimetric uncertainties for targets and normal tissues. The consequence of radiotherapy treatment on less than full bladder increases the rate of normal tissue complications. It has become essential to measure the bladder volume using cone beam CT or ultrasound prior to daily radiotherapy treatment. Assuming that the variable bladder filling volume can act as a predictor of target motion and location, pre-treatment CT scan data can be used to create a planning library. After daily verification of bladder volume, a plan of the day may be implemented for each patient. Such an adaptive treatment approach can be opted for to further decrease the dose to normal tissues.

The main limitation of our study was a relatively small number of patients, dosimetric nature of the study, and probable extra exposure to radiation for patients due to daily CBCT for checking bladder filling. In addition, the rectal filling was not taken into consideration in our study.

## Conclusion

Our study is the first reported study describing the bladder volume variations and their dosimetric impact and attempts to predict a threshold deviation of bladder filling in patients undergoing pelvic radiotherapy for intact carcinoma cervix. Bladder filling variations have an impact on cervico-uterine motion and shape, thereby impacting the dose to target and OARs. A decrease in bladder filling at the time of treatment results in an increase in dose to the OARs. However, variation in an increase in dose to OARs is not significant if bladder filling is more than 78.34% of the optimally filled bladder with respect to the bowel and 64.44% of the optimally filled bladder with respect to rectal/bladder doses. Therefore, it is recommended to have a threshold bladder volume of at least 70–75% of the initially optimally filled urinary bladder at the time of daily treatment which can be verified using a daily cone beam CT scan or ultrasonography. Online adaptive strategies can be used to select the plan of the day from the planning library prior to treatment delivery.

## Data Availability

All data generated or analyzed during this study are included in this published article.

## Abbreviations

C.I.: Conformity index
COM: Center of mass
CT simulation: Computed tomography simulation
CTRT: Chemoradiation
CTV: Clinical target volume
Dmax: Maximum dose
ECOG: Eastern Co-operative Oncology Group
GTV: Gross target volume
Gy: Gray
H.I.: Homogeneity index
IMRT: Intensity-modulated radiotherapy
OARs: Organ at risk
PET: Positron emission tomography
PLAN0: Radiotherapy planning done on empty bladder
PLAN100: Radiotherapy planning done on full bladder volume (X ml)
PLAN50: Radiotherapy planning done on 0.5X ml
PLAN70: Radiotherapy planning done on 0.7X ml
PTV: Planning target volume
RTOG: Radiation Therapy Oncology Group
SPSS: Statistical Package for the Social Sciences
TV: Target volume
*Vx*: Volume receiving “*x*” Gy dose
